# Dynamic Transcriptomic Profiling During Liver Development in *Schizothorax Prenanti*

**DOI:** 10.3389/fphys.2022.928858

**Published:** 2022-07-11

**Authors:** Jiahui Ni, Peng Zhu, Qilang Mo, Wei Luo, Zongjun Du, Jun Jiang, Song Yang, Liulan Zhao, Quan Gong, Yan Wang

**Affiliations:** ^1^ College of Animal Science and Technology, Sichuan Agricultural University, Chengdu, China; ^2^ Fisheries Institute, Sichuan Academy of Agricultural Sciences, Chengdu, China

**Keywords:** liver, development, RNA-seq, S. prenanti., metabolism

## Abstract

Liver is an important organ for glucose and lipid metabolism, immunity, and detoxification in fish. However, the gene regulatory network of postnatal liver development still remains unknown in teleost fish. In this study, we performed transcriptome analysis on the liver of *S. prenanti* at three stages. A total of 1692 differentially expressed genes (DGEs) were identified across three liver developmental stages. The oil red O staining and PAS staining revealed that the lipid content of liver was increased and the glycogen content of liver was decreased during liver development. The fatty acids biosynthesis related genes were upregulated in adult and young stages compared with juvenile stage, while lipid degradation related genes were downregulated. The genes related to glycolysis, gluconeogenesis and glycogenolysis were upregulated in juvenile or young stages compared with adult stage. Further pathway analysis indicated that the CYP450 pathway, cell cycle and amino acid metabolic pathway were induced in the process of liver maturation. Our study presents the gene expression pattern in different liver development stages of *S. prenanti* and may guide future studies on metabolism of *S. prenanti* liver.

## Introduction

The liver is an important digestive and metabolic organ of fish. Previous studies have been conducted on the effects of dietary and metabolic process on lipid deposition in liver of fish (Dai et al., 2015; Jia et al., 2020; Sun et al., 2021). It is not only an important site of glucose and lipid metabolism in fish, but also has the function of immunity and detoxification (Enes et al., 2009; Freitas-Lopes et al., 2017). Liver is mainly a hematopoietic organ during the embryonic stage, and is transformed into a major metabolic organ during the mature stage (Chapple et al., 2013; Lv et al., 2014). Wu et al. revealed that the hepatic immune functional, cell proliferation, and apoptotic related pathways are involved in postnatal liver maturation of breeder roosters (Wu et al., 2018). In addition, liver antioxidant components are decreased during liver postnatal development to protect juvenile animals from the oxygen environment and toxic stimuli (Wu et al., 2019). Previous studies have proved that there are significant changes in the expression levels of CYP450 isoforms, suggesting that different transcripts are regulated during postnatal liver maturation (Cui et al., 2012a; Peng et al., 2012). In addition, some other important factors, such as IGF2BP1 (Hammerle et al., 2013), hepatocyte growth factor (HGF) (Ishikawa et al., 2001), farniate X receptor (FXR) (Peng et al., 2017), also can regulate the process of liver development. Compared with tremendous researches on liver development of mammals, the studies about gene regulatory network of postnatal liver development still remains unknown in teleost fish.

RNA-seq is a kind of high-throughput sequencing, which is an important tool for gene expression analysis in biology (Blencowe et al., 2009). With the help of RNA-seq, there has been more insight in the field of liver development in recent years. Xin *et al.* (Wang et al., 2020) revealed that there are the similarities and differences between human and mouse liver development using RNA-seq. Besides, the mRNA abundance of transporters in liver was revealed, and the expression of liver transporters was demonstrated to both age and isoform specific (Cui et al., 2012b). The RNA-seq results of chicken liver from the prenatal to the postnatal stages indicated that antioxidant defenses pathway is activated during chicken postnatal liver mature (Xu et al., 2019).

*Schizothorax prenanti* (*S.prenanti*) is commonly known as “Ya-fish”, belonging to the *Schizothoracinae* subfamily of the *Cyprinidae* family. It is cultural symbol of Ya ‘an and popular with the breeding industry (Li et al., 2018). In our previous study, single molecule real-time sequencing is performed to generate full-length transcriptome of *S. prenanti* (Wang et al., 2022). In the present study, we obtained the overall gene expression patterns of the three stages of liver by RNA-seq technique, then identified and annotated the differentially expressed genes. These results will further deepen our understanding of the liver metabolism of *S. prenanti* in different stages, and provide a reference for the liver health of *S. prenanti* in the artificial cultivation in the future.

## Methods

### Experimental Animals and Feeding Management

The study was performed with *S. prenanti* of three different ages: 6 months (juvenile, 9.41 ± 0.60 g, *n* = 3), 1.5 years (young, 110.11 ± 10.82 g, *n* = 3), and 3 years (adult, 673.33 ± 25.17 g *n* = 3) of age, denoted as S, M, and L, respectively. All the fish were maintained for cultivation at the Fish Breeding Center of Sichuan Agriculture University (Ya’an, China) and were kept at 17 ± 1°C. Commercial fish food pellet was resupplied twice daily (9:00 a.m. and 6:00 p.m.). After acclimation for 2 weeks, healthy *S. prenanti* for normal feeding are used for experiments. Prior to sampling, fish were fasted for 24 h. Each fish was anesthetized with MS-222 (80 mg/L) and weighed. After dissection, liver was washed with PBS buffer solution, then placed in a 2 ml centrifuge tube and quickly preserved in liquid nitrogen.

### Histological Structure and Oil Red O Staining of Liver

Liver samples from three stages were fixed in 4% paraformaldehyde and sections (4 μm) were cut and stained with PAS staining. Then cryo sectioned and frozen sections were subjected to standard Oil Red O staining (Servicebio, Wuhan, China). The slides were seal-capped with glycerogelatin and were photographed by a Nikon Eclipse Ti-SR inverted microscope.

### Triglyceride and Glycogen Concentration Measurement

The contents of triglycerides were measured by Triglyceride Assay Kit (Jiancheng Biotech Co, Nanjing, China). Briefly, the liver samples treated with anhydrous ethanol were co-incubated with GPO-PAP at 37°C for 10 min, and the absorbance value was measured at 510 nm. The content of glycogen was measured by Glycogen Assay Kit (Jiancheng Biotech Co, Nanjing, China). Briefly, the glycogen detection solution was prepared, and mixed with the chromogenic solution, then incubated at 100°C for 5 min. The absorbance of samples was measured at 620 nm by Varioskan LUX Microplate Reader.

### RNA Preparation, Illumina Library Construction and Sequencing

Total RNA was extracted by the RNAiso Pure RNA Isolation Kit (TaKaRa, Tokyo, Japan). The quality of total RNA was measured by Agilent Bioanalyzer 2100 system (Agilent Technologies, CA, United States). The eukaryotic mRNA was enriched with Oligo (dT) beads by A-T complementary pairing principle. The first-strand cDNA was synthesized by random hexamers based on the mRNA template, and then the second-strand cDNA was synthesized by adding buffer, DNTPs, RNase H and DNA Polymerase I. Then the double-stranded cDNA was purified and a-tailed. After that, sequencing adaptors were attached and the fragment size was selected by AMPure XP beads. Finally, the cDNA library was obtained by PCR and sequenced on the Illumina NovaSeq 6000 platform.

### Identification and Expression Analysis of Differentially Expressed Genes

Clean reads were obtained after removing ploy-N and low-quality reads from raw data. Then, the base quality of the clean reads was evaluated by GC content and Q30. The clean reads were mapped onto transcripts of full-length transcriptome using the RNA-seq comparison software STAR (Dobin et al., 2013). We aligned the transcript sequence to NR (Deng et al., 2006), Swissprot (Rolf et al., 2004), GO (Ashburner et al., 2000), COG (Tatusov et al., 2000), KOG (Koonin et al., 2004), Pfam (Finn et al., 2014), KEGG (Minoru et al., 2004) databases by BLAST software (version 2.2.26) to obtain the annotation information of the transcript. The FPKM distribution density was quantified using RSEM (Li and Dewey, 2011). Subsequently, DESeq software package was used to analyze differentially expressed genes, which could remove genes that were not expressed (counts = 0) at least in two samples (Anders and Huber, 2010), and then the *p*-values were adjusted for controlling the false discovery rate (FDR) by the Benjamini and Hochberg method (Storey and Tibshirani, 2003). The screening criteria were fold change ≥2 and FDR <0.05. KOBAS 2.0 software was used to test the statistical enrichment of differentially expressed genes in KEGG pathways (Xie et al., 2011).

### Quantitative Real-Time PCR

The cDNA synthesis was used by HiScript^®^ III RT SuperMix for qPCR (Vazyme, Nanjing, China). CFX96TM Real-Time PCR Detection System (Bio-Rad, United States) was used to perform qPCR in a final volume of 10 μl: 3.8 μl of sterilized double-distilled water, 5 μl of SYBR qPCR Master, 0.8 μl of cDNA and 0.4 μl of each primer. The primers as shown in Supplementary Table S1. Transcript levels were normalized to the expression of the reference gene β-actin by the 2^−ΔΔCt^ method.

### Enzyme Assay

The activity of PEPCK and PFK was determined with Phosphoenolpyruvate Carboxykinase Activity Assay Kit (Jiancheng Biotech Co, Nanjing, China) and Phosphofructokinase Activity Assay Kit (Jiancheng Biotech Co, Nanjing, China), respectively. The mixture of 0.1 g tissue and 1 ml extraction solution was used for ice bath homogenization. Take the upper part and put it on ice for test. Then we added the reagent and sample into 1 ml quartz cuvette according to the instructions, and read the absorbance at 340 nm by a UV-2800 spectrophotometer (BMS Biotechnology Medical Services, Madrid, Spain).

### Statistical Analysis

All data are expressed as the mean ± SEM. The statistical analyses of one-way ANOVA were performed using SPSS 19.0 (IBM, NY, United States). All data were represented as mean ± SEM. And groups denoted by different letters represent a significant difference at *p* < 0.05.

## Results

### Illumina RNA-Seq Quality Validation

**FIGURE 1:**
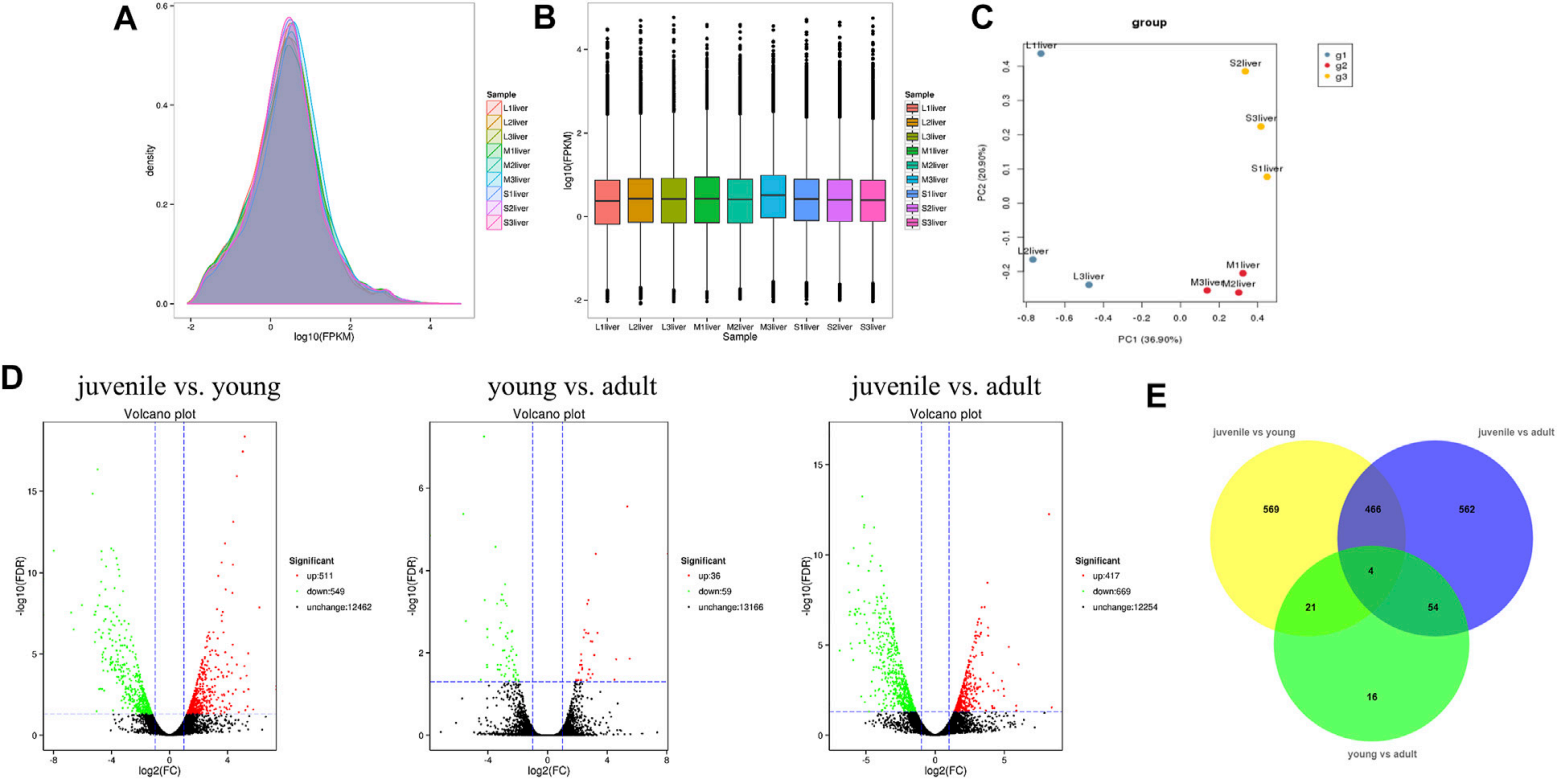
Quality assessment and analysis of global gene expression among liver samples at three stages. **(A)** Intuitive display genes expressed at different FPKM levels. **(B)** Boxplot of FPKM distribution among liver samples. **(C)** Principal component analysis (PCA) plots of all transprits at diffierent stages in S. prenanti. **(D)** Volcano plot of differential expression genes among the three stages in the pairwise comparisons. **(E)** Venn diagram showing the number of DEGs in transcript abundance in liver of *S. prenanti*. Yellow circles indicate the samples of juvenile vs young, green circles indicate the samples of young vs adult, and blue circles indicate samples of juvenile vs adult.

To identify the difference of gene expression in different stages of *S. prenanti,* a total of 9 liver cDNA libraries in three periods were constructed. After quality control of sequencing data, a total of 57.22G clean data were obtained, with Q30 reaching more than 85% (Table 1). Alignment results of clean reads and transcripts are presented in Table 2. Normalize the number of reads mapped to all genes and calculate it as FPKM for evaluating gene expression. The FPKM distribution density displayed the overall distribution of different transcripts expression levels in 9 liver samples (Figure 1A). The dispersion degree of the different samples was also generated with box plots (Figure 1B), which measured the expression level of each sample from the overall discrete expression level. The PCA score plot showed that the three stages were clearly separated with the main principal component (PC) scores as follows: PC1 = 36.90%, PC2 = 20.90% (Figure 1C).

**TABLE 1:** Statistical table for sample sequencing data evaluation.

Samples	BMK-ID	Read Number	Base Number	GC Content	%≥Q30
Adult1liver	L1liver	21,498,046	6,408,391,356	47.28	93.66
Adult2liver	L2liver	21,224,260	6,345,019,826	47.85	93.84
Adult3liver	L3liver	21,873,325	6,534,813,036	47.98	93.83
Young1liver	M1liver	20,927,905	6,250,744,092	47.57	93.69
Young2liver	M2liver	21,009,982	6,281,676,604	48.49	93.84
Young3liver	M3liver	21,748,283	6,483,993,762	47.68	93.07
Juvenile1liver	S1liver	21,527,874	6,433,382,690	47.95	92.97
Juvenile2liver	S2liver	21,070,071	6,291,554,182	48.2	92.87
Juvenile3liver	S3liver	20,741,711	6,195,120,812	48.27	93.1

**TABLE 2:** Statistical table of comparison results between the second-generation sequencing data and the third-generation non-redundant transcripts.

Sample	Total Reads	Uniquely Mapped reads (%)	% of reads Mapped to Multiple Loci	% of reads Mapped to too Many Loci
Adult1liver	21,498,046	44.61	27.32	0.03
Adult2liver	21,224,260	49.83	24.67	0.04
Adult3liver	21,873,325	49.92	24.47	0.04
Young1liver	20,927,905	49.55	21.65	0.08
Young2liver	21,009,982	51.26	22.84	0.09
Young3liver	21,748,283	46.27	21.77	0.05
Juvenile1liver	21,527,874	53.07	20.02	0.05
Juvenile2liver	21,070,071	52.05	21.27	0.04
Juvenile3liver	20,741,711	52.06	20.41	0.04

### Analysis of Differentially Expressed Genes

A total of 1692 DEGs were detected across three liver developmental stages (Supplementary Table S2). The expression levels of genes among the three groups in the pairwise comparisons were viewed through the Volcano Plot. As shown in Figure 1D, the highest number of DEGs was found between juvenile and young fish, including 511 upregulated and 549 downregulated genes. Between the juvenile and adult fish, 417 genes were upregulated and 669 genes were downregulated. There were only 95 DEGs between the young and adult fish, including 36 upregulated and 59 downregulated genes. A Venn diagram was constructed to identify the joint DEGs among three pair-wise stage comparisons. Through Venn diagram analysis, we identified 470 DEGs that were co-altered in both juvenile vs. young and juvenile vs. adult. A total of 25 and 58 DEGs presented the same expressed trends between juvenile vs. young and young vs. adult, young vs. adult and juvenile vs. adult, respectively, (Figure 1E).

### GO and KEGG Pathway Enrichment Based on DEGs

**FIGURE 2:**
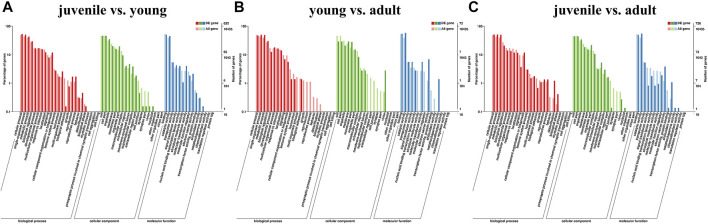
Classification of different expression genes (DEG) with GO databasesa. **(A)** juvenile vs. young, **(B)** young vs. adult, **(C)** juvenile vs. adult. The abscissa is the GO classification, the left of the ordinate is the percentage of transcripts, and the right is the number of transcripts. The two vertical axes are the enrichment of GO secondary functions of differentially expressed transcripts and all transcripts, respectively.

To explore the pathway of differential gene enrichment in different periods, the sequences were annotated by GO and KEGG databases. GO enrichment analysis was conducted on DEGs identified in three stages, and the results were divided into three categories: biological process (BP), cellular component (CC) and molecular function (MF) (Figures 2A–C). GO enrichment analysis showed that the major category represented was cellular process (GO:0009987) among biological processes. And among molecular functions, the major categories were catalytic activity (GO:0003824).

**FIGURE 3:**
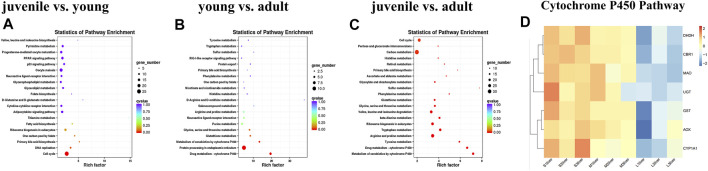
KEGG analysis of differentially expressed genes during liver development. **(A)** juvenile vs. young, **(B)** young vs. adult, **(C)** juvenile vs. adult, **(D)** heatmaps of the DEGs involved in metabolism of xenobiotics by cytochrome P450 and drug metabolism—cytochrome P450. The size of each point represents the degree of enrichment, and the color of each point represents the size of the *q*-value. Data are shown as means ± SEM. Three biological replicates were used.

Finally, KEGG pathways databases were performed to determine the enrichment of differentially expressed genes. The top 20 pathways of DEGs enrichment can be seen in Supplementary Table S3 between different stages. In the comparison of young vs. adult, only the cell cycle pathway was significantly enriched for the differentially expressed genes (Figure 3A). Comparing juvenile vs. young, the differentially expressed genes were significantly enriched in three pathways, including drug metabolism-cytochrome P450, protein processing in endoplasmic reticulum, metabolism of xenobiotics by cytochrome P450 (Figure 3B). In contrast, a number of pathways were significantly enriched for juvenile vs. adult. The top 20 KEGG pathways were shown in Figure 3C, including drug metabolism-cytochrome P450, metabolism of xenobiotics by cytochrome P450, carbon metabolism, tyrosine metabolism, arginine and proline metabolism, tryptophan metabolism. We used heatmaps to characterize the DEGs in the enriched pathway drug metabolism-cytochrome P450. The mRNA expression levels of the CYP450 pathway related genes such as *GST*, *UGT*, *DHDH*, *CBR1*, *AOX*, *MAO* and CYP450 subtypes *CYP1a1* were lower level in adult stage compared with juvenile and young stages (Figure 3D).

### Genes Involved in Lipid Metabolism

**FIGURE 4:**
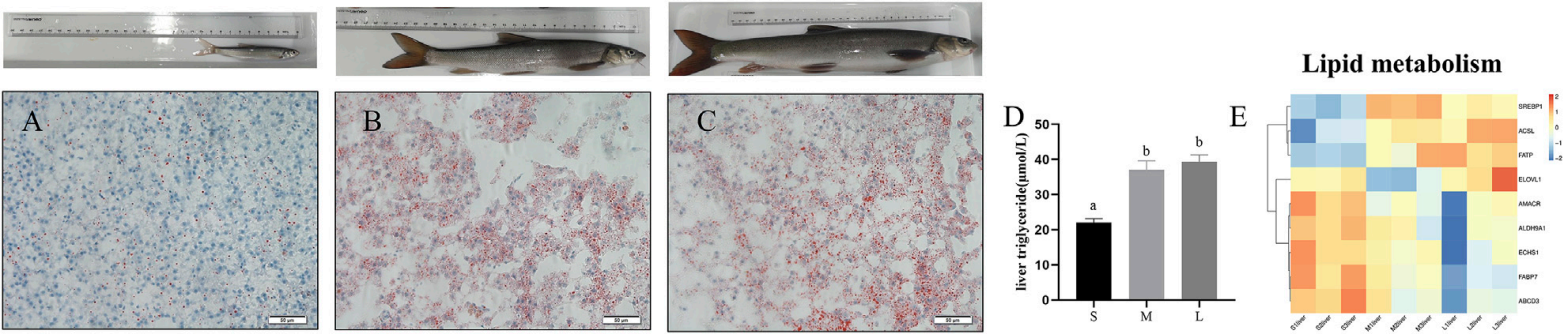
Oil red staining and TGs content in liver of *S. prenanti* at three stages. **(A)** liver oil red O staining of juvenile, **(B)** liver oil red O staining of young, **(C)** liver oil red O staining of adult, **(D)** the content of liver triglyceride, **(E)** heatmaps of the DEGs involved in fatty acid biosynthesis, fatty acid elongation, biosynthesis of unsaturated fatty acids, fatty acid degradation and PPAR signaling pathway. The juvenile, young and adult of *S. prenanti* denoted as S, M, and L, respectively. Data are shown as means ± SEM. Three biological replicates were used. Data are shown as means ± SEM. Three biological replicates were used. And groups denoted by different letters represent a significant difference at *p* < 0.05.

To investigate the contents of triacylglycerol among three stages, we performed oil red O staining to the liver section. As shown in Figures 4A–C, lipid accumulation was remarkedly increased in the liver of young and adult stages compared with juvenile stage. Meanwhile, the content of liver triglyceride was remarkedly increased at young and adult stages compared with juvenile stage (Figure 4D) (*p* < 0.05). Expression levels of genes involved in the pathways associated to fatty acid metabolism were showed in Figure 4E. Compared with juvenile stage, the mRNA expression levels of fatty acid synthesis related genes such as *FATP*, *ACSL*, *SREBP1*, *ELOVL1* were upregulated in adult and young stages, while expression of fatty acid β oxidation related genes such as *ALDH9A1*, *AMACR*, *ECHS1*, and *HADHA* were downregulated in young and adult stage compared with juvenile (Figure 4E).

### Genes Involved in Carbohydrate Metabolism

**FIGURE 5:**
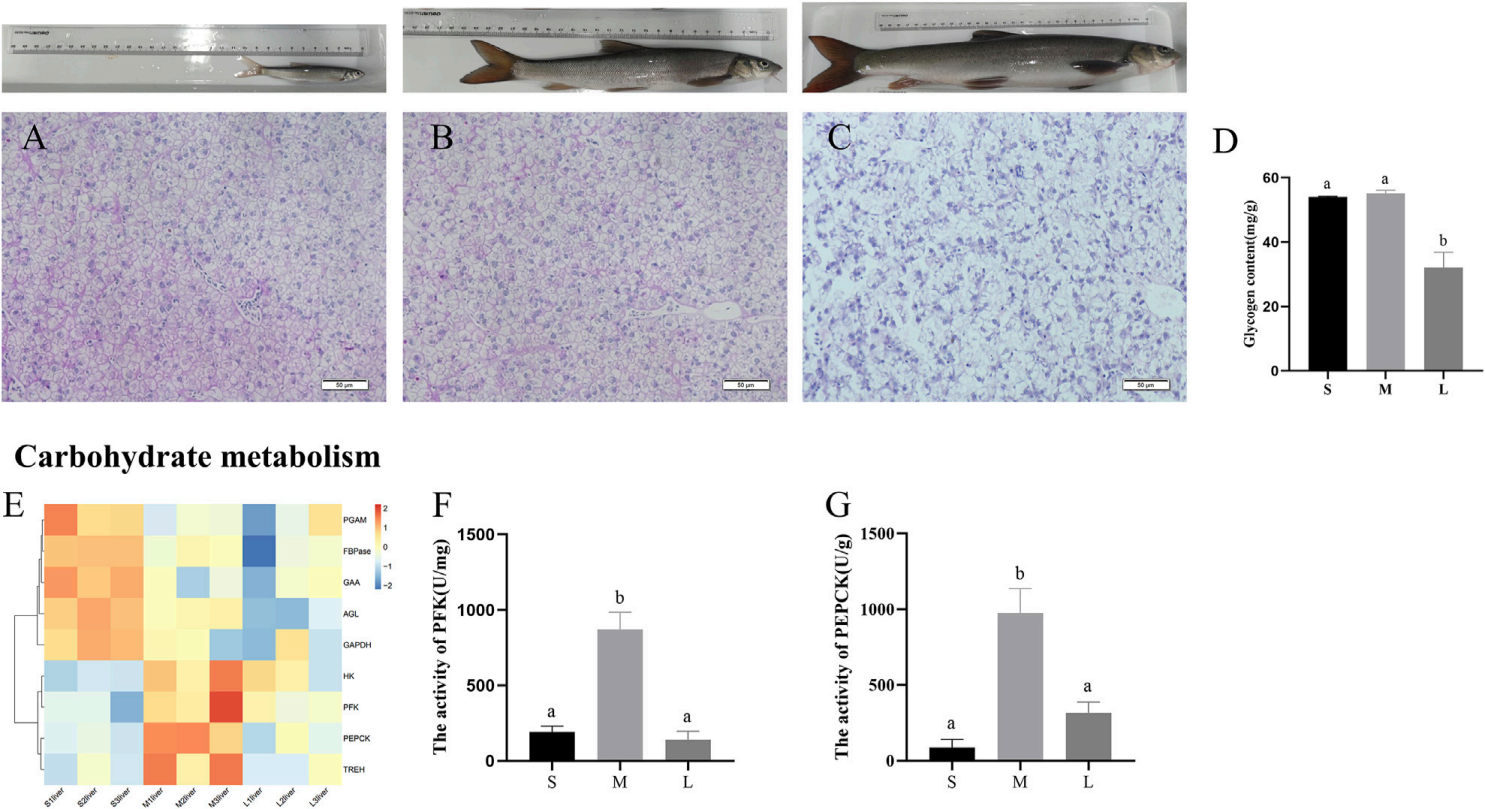
PAS staining and glycogen content in liver of *S. prenanti* at three stages. **(A)** liver PAS staining of juvenile. **(B)** liver PAS staining of young, **(C)** liver PAS staining of adult, **(D)** the content of liver glycogen, **(E)** heatmaps of the DEGs involved in glycolysis and gluconeogenesis, **(F)** The activity of PFK (U/mg), **(G)** The activity of PEPCK (U/g). Data are shown as means ± SEM. Three biological replicates were used. And groups denoted by different letters represent a significant difference at *p* < 0.05. The juvenile, young and adult of *S. prenanti* denoted as S, M, and L, respectively.

To investigate the contents of glycogen during three stages, we performed PAS staining to the liver section. As shown in Figures 5A–D, glycogen content was higher in the livers of juvenile and young stages, compared with adult stage (*p* < 0.05). Expression levels of genes involved in the main pathways associated to carbohydrate are given in Figure 5E. The pathways include glycolysis/gluconeogenesis, starch and sucrose metabolism, fructose and mannose metabolism. The genes related to glycolysis (*HK*, *PFK*, *GAPDH*, and *PGAM*), gluconeogenesis (*PEPCK* and *FBPase*) and glycogenolysis (*AGL*, *UGP2*, *GAA*, and *TREH*) were upregulated in juvenile or young stages compared with adult stage. Moreover, the activity of PFK and PEPCK was at the lowest level in juvenile and then increased to a peak in young and was downregulated from young to adult (Figures 5F,G).

### Validation of Differential Gene Expression By qPCR

**FIGURE 6:**
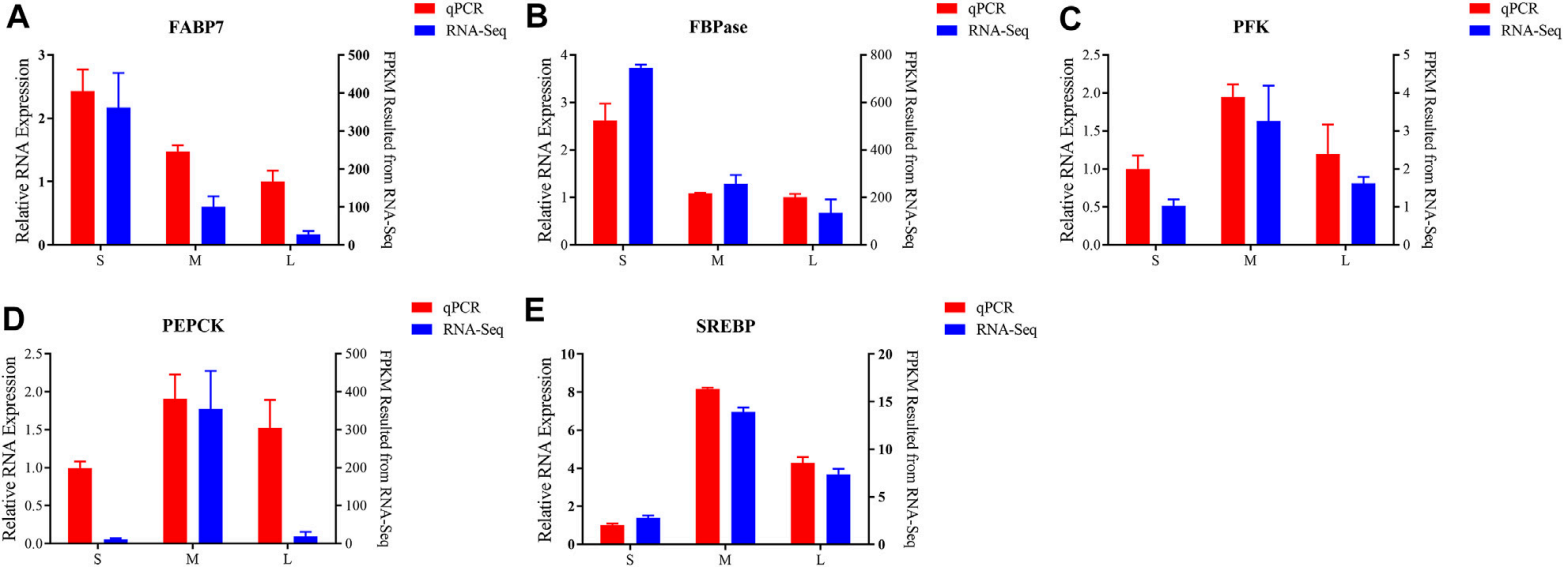
Validation of six differentially expressed of FABP7 **(A)**, FBPase **(B)**, PFK **(C)**, PEPCK **(D)**, SREBP **(E)** by qPCR. Left *y* axis shows the relative expression levels of genes using qPCR, and the right *y* axis shows the fragments per kb per million reads (FPKM) values of the using RNA-seq. Data are shown as means ± SEM. Three biological replicates were used.

To validate the accuracy of the transcriptome analysis, five differentially expression genes were selected for qPCR. As shown in Figures 6A,B, the mRNA expression levels of *FABP7* and *FBPase* were downregulated from juvenile to adult. *PFK*, *PEPCK* and *SREBP* genes were expressed at the lowest level in juvenile and then increased to a peak in young, and was downregulated from young to adult (Figures 6C–E). As the qPCR results shown, the expression of all the tested genes presented the same expressed trends with the results of transcriptome analysis, suggesting that the results of RNA-seq were accurate and credible.

## Discussion

In vertebrates, the liver is the main place for metabolic clearance of foreign compounds, and is the richest source of drug metabolizing enzymes (Parkinson et al., 2001). In this study, the differentially expressed genes were significantly enriched in drug metabolism-cytochrome P450 and metabolism of xenobiotics by cytochrome P450 pathways. Drug metabolic enzyme can be divided into cytochrome P450 enzymes and non-cytochrome P450 enzymes. CYP450 is generated by liver microsomes, which is an important enzyme in the metabolism of environmental pollutants and endogenous substances (Waxman, 1999). The expression and activity of CYP isoforms in liver alter significantly with age during development, and CYP1A1 is detected only in 3-week-old rats (Yun et al., 2010). In rainbow trout, the concentration of CYP450 is negatively correlated with body weight in the process of growing (Fitzsimmons et al., 2018). In the present study, the mRNA expression of CYP450 subtypes *CYP1a1* were lower level in adult stage compared with juvenile and young stage.

Non-cytochrome P450 enzymes includes DHDH, CBR1, GST, MAO, AOX, UGT, and so on (Pang et al., 2022). Dihydrodiol dehydrogenase (DHDH) is involved in the metabolism of polycyclic aromatic hydrocarbons in the liver (Carbone et al., 2008), but there is no report on the development change of DHDH in teleost fishes. In the present study, the mRNA expression of *DHDH* were lower level in adult stage compared with juvenile and young stage. The activity of GST is elevated at 24 h under the action of low concentration toxicity (triclosan), significantly inhibited at 72 h, but induced at 168 h in yellow catfish (Ku et al., 2014). In addition, Neta et al. have proved that GST activity is changed in different periods in catfish sampled (Neta et al., 2017). Previous study showed that the activities of CBR1 and GST are 2.4 folds and 5.6 folds higher in the 21-month-old rats compared with 6-week-old rats, respectively, but UGT is significantly decreased (Vyskocilova et al., 2013). Carbonyl reductase 1 (CBR1) participates the biotransformation of various xenobiotics containing carbonyl group. In this study, the expression of *GST*, *UGT*, and *CBR1* were decreased, suggesting that the ability of the biotransformation of various xenobiotics in the liver becomes lower during liver development. Monoamine oxidase (MAO) and aldehyde oxidase (AOX) are enzymes that catalyzes the oxidation of various drugs and endogenous compounds (Petrovic et al., 1991; Tipton et al., 2004; Tayama et al., 2007). The expression levels of MAO-A and MAO-B decreased in the liver of aged mice (Saura et al., 1994). Unlike mammals, which have two types of MAO (MAO-A and MAO-B), there is only one single form of MAO in fish (Nicotra et al., 2004). AOX activity in rats is increased rapidly from birth and reached a plateau within 4 weeks, which is connected with postnatal liver development of mice (Tayama et al., 2007). In fish, there are few studies on these enzymes of CYP450 pathway. In this study, the expression of drug metabolism-cytochrome P450 related genes were decreased during the liver development of *S. prenanti*, suggesting that the oxidation ability of various drugs and endogenous compounds may be inhibited in adult comparing with juvenile and young stages.

Liver is one of the important sites of amino acid metabolism. Amino acids play crucial roles on the growth, reproduction, and immune responses in fish (Li et al., 2009). Arginine and proline are amino acids with crucial roles in protein deposition and the immune response (Wu et al., 2011). In addition, dietary tryptophan attenuated stress induced anorexia and reduce aggressive behavior in brown trout (Andersen et al., 2016). Arginine and proline metabolism pathway and tryptophan metabolism pathway were significantly enriched during chicken liver development (Wu et al., 2018). Consistent with this, arginine and proline metabolism pathway and tryptophan metabolism pathway were significantly enriched based on the DEGs, which suggested that amino acids pathway was involved in development of *S. prenanti*.

Lipid synthesis and fatty acid β oxidation play a critical role in lipid deposition of liver. SREBP1 is a key transcriptional factor of lipid synthesis, regulating expression of fatty acid and triglyceride synthesis related genes (Jeon and Osborne, 2012). ACSLs catalyze the synthesis of acyl CoA from fatty acids, which is the key enzyme of triglyceride synthesis and fatty acid β oxidation (Grevengoed et al., 2014). Our results revealed that the lipid content of liver increased in adult and young stages comparing with juvenile. Consistent with the oil-red O staining results, the mRNA expression levels of *SREBP1* and *ACSLs* increased in young and adult stage compared with juvenile, indicating the fatty acid and triglyceride synthesis maybe active in young and adult stage. Previous studies proved that *ELOVL1* can elongate very long chain monounsaturated fatty acid and very long-chain saturated fatty acids (Moon et al., 2009; Ohno et al., 2010). In the present study, the mRNA expression of *ELOVL1* was increased during liver development, which suggested that liver fatty acid elongation may be induced during the liver development. FATP is a key factor involved in fatty acid transport and fat deposition, which function is to transport of long-chain and very long chain fatty acids into cells (Dutta-Roy, 2000). In the conditions of low-fat feeding, the expression level of FATP is upregulated in the liver of 18 months mice comparing to 3 months mice (Martin et al., 2008). In our RNA-seq results, we found that the mRNA expression of *FATP* was also increased in young and adult stage comparing with juvenile. *FABP* can bind fatty acids and transport them to peroxisome, mitochondria and endoplasmic reticulum for fatty acid β oxidation (Veerkamp et al., 1993). In mice, *L-FABP* gene ablation increases the concentration of liver lipid concentrations (Martin et al., 2008). In the resent study, mRNA expression of *FABP* was the lowest in adult of the three periods. Meanwhile, the mRNA expression of fatty acid β oxidation related genes including *ALDH9A1*, *AMACR*, *ECHS1*, *HADHA* were consistent with the results of FABP, which suggested that fatty acid β oxidation pathway was downregulated in adult stage. Collectively, the results revealed that the pathways of lipid synthesis and fatty acid elongation was elicited, and fatty acid β oxidation pathway was downregulated in the liver development.

Liver is the main site of carbohydrate metabolism in fish. Our PAS staining revealed that the glycogen content of liver decreased during liver devepment. Hexokinase (HK) and phosphofructokinase (PFK) are the rate-limiting enzymes of glycolysis. In rats, hexokinase activity has no significant change during liver postnatal development (Dileepan et al., 1979). In our study, mRNA expression levels encoding these two enzymes and the activity of PFK were the highest in young stage, suggesting that glycolysis ability may be enhanced in young stage. PEPCK and FBPase are the rate-limiting enzymes of gluconeogenesis, and catalyzes oxaloacetic acid to form phosphoenolpyruvate and the conversion of fructose-1, 6-diphosphate to fructose-6-phosphate (El-Maghrabi et al., 1995; Hanson, 2009). We found that the mRNA expression of *PEPCK* and *FBPase* as well as the activity of PEPCK were decreased at the adult stage, which indicated that the gluconeogenesis pathway was inhibited in adult stage. Glycogen degradation is catalyzed by many enzymes, which including AGL, GAA and TREH (Yong et al., 1996; Lombard et al., 2014). In this study, the mRNA of these genes showed that glycogenolysis decreased in adulthood. These results indicated that glycolysis, gluconeogenesis and glycogenolysis pathway were inhibited during liver development of *S. prenanti*. With the growth of individuals, the demand for feed lipid and carbohydrate varies at different stages. In fish, many studies have confirmed that high carbohydrate and high fat diets can lead to the increase of lipid accumulation and glycogen content in fish liver (Prisingkorn et al., 2017; Jia et al., 2020; Sun et al., 2021). Therefore, suitable nutrient levels are able to affect fish health and promote fish growth. Previous study showed that fatty acid deficiencies manifest themselves faster in juvenile fish (Dabrowski, 1986). For example, essential fatty acid (EFA) deficiency occurs only within 2 weeks when young juvenile barramundis (*Lates calcarifer*) are fed with fish oil free diet, or with a low inclusion of fish oil (Salini et al., 2015). In seabass (*Dicentrarchus labrax*) juveniles, reducing dietary fish oil levels from 6% to 3% increases gut bacterial translocation rates (Torrecillas et al., 2017). In this study, lipid accumulation was remarkedly increased in the liver of young and adult stages compared with juvenile stage, and we speculated that the crude lipid requirement of juvenile is higher than that of young and adult. In gilthead sea bream (*Sparus aurata*) juveniles, the growth performance, feed efficiency, and protein efficiency ratio of 30% gelatinized maize starch were lower than those of 20 and 10% gelatinized maize starch fish fed diet (Couto et al., 2008). In this study, glycogen content was higher in the livers of juvenile and young stages, compared with adult stage, and we speculated that the carbohydrate requirement of juvenile and young is lower than that of adult.

In this study, we obtained mRNA expression patterns of three stages in *S. prenanti* liver by RNA-seq technology and have identified a total of 1692 differentially expressed mRNAs across three stages of *S. prenanti* liver. In addition, we revealed that lipid accumulation was increased and glycogen content was decreased during liver development. Moreover, based on KEGG analysis we found that the differentially expressed mRNAs were involved in the CYP450 pathway, lipid metabolism and carbohydrate metabolism in liver maturation of *S. prenanti*. Overall, our data enriched sequences information of *S. prenanti* and provided a broad and novel vision for future research at the transcription level in fish.

## Data Availability

The datasets presented in this study can be found in online repositories. The names of the repository/repositories and accession number(s) can be found in the article/Supplementary Material.

## Abbreviations

Aldehyde dehydrogenase family 9 member A1, ALDH9A1; alpha-methylacyl-CoA racemase, AMACR; Aldehyde oxidase, AOX; 2,3-bisphosphoglycerate-dependent phosphoglycerate mutase, PGAM; Carbonyl reductase 1, CBR1; Dihydrodiol dehydrogenase, DHDH; Elongation of very long chain fatty acids protein 1, ELOVL1; Enoyl-CoA hydratase, ECHS1; Enoyl-CoA hydratase, HADHA; Fatty acid transporter, FATP; Fatty Acid Binding Protein 1, FABP; Fructose-1,6-bisphosphatase, FBPase; Glycogen debranching enzyme, AGL; Glyceraldehyde 3-phosphate dehydrogenase, GAPDH; Glucuronosyltransferase, UGT; Hexokinase, HK; Lysosomal alpha-glucosidase, GAA; Long-chain acyl-CoA synthases, ACSLs; Monoamine oxidase, MAO; 6-phosphofructokinase 1, PFK; Phosphoenolpyruvate carboxykinase, PEPCK; Sterol regulatory element binding protein, SREBP1; UTP--glucose-1-phosphate uridylyltransferase, UGP2.

## References

[B1] Anders S. Huber W. (2010). Differential Expression Analysis for Sequence Count Data. Genome Biol. 11 (10), R106. 10.1186/gb-2010-11-10-r106 20979621 PMC3218662

[B2] Ashburner M. Ball C. A. Blake J. A. Botstein D. Butler H. Cherry J. M. (2000). Gene Ontology: Tool for the Unification of Biology. Nat. Genet. 25 (1), 25–29. 10.1038/75556 10802651 PMC3037419

[B3] Blencowe B. J. Ahmad S. Lee L. J. (2009). Current-generation High-Throughput Sequencing: Deepening Insights into Mammalian Transcriptomes. Genes Dev. 23 (12), 1379–1386. 10.1101/gad.1788009 19528315

[B4] Carbone V. Hara A. El-Kabbani O. (2008). Structural and Functional Features of Dimeric Dihydrodiol Dehydrogenase. Cell. Mol. Life Sci. 65 (10), 1464–1474. 10.1007/s00018-008-7508-5 18264804 PMC11131880

[B5] Chapple R. H. Tizioto P. C. Wells K. D. Givan S. A. Kim J. McKay S. D. (2013). Characterization of the Rat Developmental Liver Transcriptome. Physiol. Genomics 45 (8), 301–311. 10.1152/physiolgenomics.00128.2012 23429212 PMC3633428

[B6] Couto A. Enes P. Peres H. Oliva-Teles A. (2008). Effect of Water Temperature and Dietary Starch on Growth and Metabolic Utilization of Diets in Gilthead Sea Bream (*Sparus aurata*) Juveniles. Comp. Biochem. Physiology Part A Mol. Integr. Physiology 151 (1), 45–50. 10.1016/j.cbpa.2008.05.013 18586542

[B7] Cui J. Y. Gunewardena S. S. Yoo B. Liu J. Renaud H. J. Lu H. (2012b). RNA-seq Reveals Different mRNA Abundance of Transporters and Their Alternative Transcript Isoforms during Liver Development. Toxicol. Sci. 127 (2), 592–608. 10.1093/toxsci/kfs107 22454430 PMC3355312

[B8] Cui J. Y. Renaud H. J. Klaassen C. D. (2012a). Ontogeny of Novel Cytochrome P450 Gene Isoforms during Postnatal Liver Maturation in Mice. Drug Metab. Dispos. 40 (6), 1226–1237. 10.1124/dmd.111.042697 22446519 PMC3362787

[B9] Dabrowski K. R. (1986). Ontogenetical Aspects of Nutritional Requirements in Fish. Comp. Biochem. Physiology Part A Physiology 85 (4), 639–655. 10.1016/0300-9629(86)90272-0 2879670

[B10] Dai W. Wang K. Zheng X. Chen X. Zhang W. Zhang Y. (2015). High Fat Plus High Cholesterol Diet Lead to Hepatic Steatosis in Zebrafish Larvae: a Novel Model for Screening Anti-hepatic Steatosis Drugs. Nutr. Metab. (Lond) 12, 42. 10.1186/s12986-015-0036-z 26583037 PMC4650307

[B11] Deng Y. Jianqi L. I. Songfeng W. U. Zhu Y. Chen Y. Fuchu H. E. J. C. E. (2006). Integrated Nr Database in Protein Annotation System and its Localization. 32(5)**,** 71–72.

[B12] Dileepan K. N. Wagle S. R. Hofmann F. Decker K. (1979). Distribution Profile of Glucokinase and Hexokinase in Parenchymal and Sinusoidal Cells of Rat Liver during Development. Life Sci. 24 (1), 89–95. 10.1016/0024-3205(79)90284-4 763072

[B13] Dobin A. Davis C. A. Schlesinger F. Drenkow J. Zaleski C. Jha S. (2013). STAR: Ultrafast Universal RNA-Seq Aligner. Bioinformatics 29 (1), 15–21. 10.1093/bioinformatics/bts635 23104886 PMC3530905

[B14] Dutta-Roy A. K. (2000). Cellular Uptake of Long-Chain Fatty Acids: Role of Membrane-Associated Fatty-Acid-Binding/transport Proteins. CMLS, Cell. Mol. Life Sci. 57 (10), 1360–1372. 10.1007/PL00000621 11078015 PMC11146826

[B15] El-Maghrabi M. R. Lange A. J. Jiang W. Yamagata K. Stoffel M. Takeda J. (1995). Human Fructose-1,6-Bisphosphatase Gene (FBP1): Exon-Intron Organization, Localization to Chromosome Bands 9q22.2-q22.3, and Mutation Screening in Subjects with Fructose-1,6-Bisphosphatase Deficiency. Genomics 27 (3), 520–525. 10.1006/geno.1995.1085 7558035

[B16] Enes P. Panserat S. Kaushik S. Oliva-Teles A. (2009). Nutritional Regulation of Hepatic Glucose Metabolism in Fish. Fish. Physiol. Biochem. 35 (3), 519–539. 10.1007/s10695-008-9259-5 18791853

[B17] Espe M. Waagbo R. Espe M. (2016). Functional Amino Acids in Fish Nutrition Health and Welfare. Front. Biosci. 8 (1), 143–169. 10.2741/757 26709652

[B18] Finn R. D. Bateman A. Clements J. Coggill P. Eberhardt R. Y. Eddy S. R. (2014). Pfam: the Protein Families Database. Nucl. Acids Res. 42 (D1), D222–D230. 10.1093/nar/gkt1223 24288371 PMC3965110

[B19] Fitzsimmons P. N. Hoffman A. D. Fay K. A. Nichols J. W. (2018). Allometric Scaling of Hepatic Biotransformation in Rainbow Trout. Comp. Biochem. Physiology Part C Toxicol. Pharmacol. 214, 52–60. 10.1016/j.cbpc.2018.08.004 PMC6349251 30172734

[B20] Fortes Carvalho Neta R. N. Barbosa G. L. Torres H. S. Pinheiro Sousa D. B. Castro J. d. S. Santos D. M. S. (2017). Changes in Glutathione S-Transferase Activity and Parental Care Patterns in a Catfish (Pisces, Ariidae) as a Biomarker of Anthropogenic Impact in a Brazilian Harbor. Arch. Environ. Contam. Toxicol. 72 (1), 132–141. 10.1007/s00244-016-0326-0 27864585

[B21] Freitas-Lopes M. Mafra K. David B. Carvalho-Gontijo R. Menezes G. (2017). Differential Location and Distribution of Hepatic Immune Cells. Cells 6 (4), 48. 10.3390/cells6040048 PMC5755505 29215603

[B22] Grevengoed T. J. Klett E. L. Coleman R. A. (2014). Acyl-CoA Metabolism and Partitioning. Annu. Rev. Nutr. 34, 1–30. 10.1146/annurev-nutr-071813-105541 24819326 PMC5881898

[B23] Hämmerle M. Gutschner T. Uckelmann H. Ozgur S. Fiskin E. Gross M. (2013). Posttranscriptional Destabilization of the Liver-specific Long Noncoding RNA HULC by the IGF2 mRNA-Binding Protein 1 (IGF2BP1). Hepatology 58 (5), 1703–1712. 10.1002/hep.26537 23728852

[B24] Hanson R. W. (2009). Thematic Minireview Series: a Perspective on the Biology of Phosphoenolpyruvate Carboxykinase 55 Years after its Discovery. J. Biol. Chem. 284 (40), 27021–27023. 10.1074/jbc.R109.040519 19636078 PMC2785630

[B25] Ishikawa K. S. Masui T. Ishikawa K. Shiojiri N. (2001). Immunolocalization of Hepatocyte Growth Factor and its Receptor (C-Met) during Mouse Liver Development. Histochem Cell Biol. 116 (5), 453–462. 10.1007/s00418-001-0342-6 11735009

[B26] Jeon T.-I. Osborne T. F. (2012). SREBPs: Metabolic Integrators in Physiology and Metabolism. Trends Endocrinol. Metabolism 23 (2), 65–72. 10.1016/j.tem.2011.10.004 PMC3273665 22154484

[B27] Jia R. Cao L.-P. Du J.-L. He Q. Gu Z.-Y. Jeney G. (2020). Effects of High-Fat Diet on Antioxidative Status, Apoptosis and Inflammation in Liver of tilapia (*Oreochromis niloticus*) via Nrf2, TLRs and JNK Pathways. Fish Shellfish Immunol. 104, 391–401. 10.1016/j.fsi.2020.06.025 32553566

[B28] Koonin E. V. Fedorova N. D. Jackson J. D. Jacobs A. R. Krylov D. M. Makarova K. S. (2004). A Comprehensive Evolutionary Classification of Proteins Encoded in Complete Eukaryotic Genomes. Genome Biol. 5 (2), R7. 10.1186/gb-2004-5-2-r7 14759257 PMC395751

[B29] Ku P. Wu X. Nie X. Ou R. Wang L. Su T. (2014). Effects of Triclosan on the Detoxification System in the Yellow Catfish (*Pelteobagrus fulvidraco*): Expressions of CYP and GST Genes and Corresponding Enzyme Activity in Phase I, II and Antioxidant System. Comp. Biochem. Physiology Part C Toxicol. Pharmacol. 166, 105–114. 10.1016/j.cbpc.2014.07.006 25064140

[B30] Li B. Dewey C. N. (2011). RSEM: Accurate Transcript Quantification from RNA-Seq Data with or without a Reference Genome. BMC Bioinforma. 12, 323. 10.1186/1471-2105-12-323 PMC3163565 21816040

[B31] Li P. Mai K. Trushenski J. Wu G. (2009). New Developments in Fish Amino Acid Nutrition: towards Functional and Environmentally Oriented Aquafeeds. Amino Acids 37 (1), 43–53. 10.1007/s00726-008-0171-1 18751871

[B32] Li Y. Wu J. Li D. Huang A. Bu G. Meng F. (2018). Teleost-specific TLR25 Identified from Schizothorax Prenanti May Recognize Bacterial/viral Components and Activate NF-Κb and Type I IFNs Signaling Pathways. Fish. Shellfish Immunol. 82, 361–370. 10.1016/j.fsi.2018.08.007 30081181

[B33] Lombard V. Golaconda Ramulu H. Drula E. Coutinho P. M. Henrissat B. (2014). The Carbohydrate-Active Enzymes Database (CAZy) in 2013. Nucl. Acids Res. 42 (Database issue), D490–D495. 10.1093/nar/gkt1178 24270786 PMC3965031

[B34] Lv J. Huang Z. Liu H. Liu H. Cui W. Li B. (2014). Identification and Characterization of Long Intergenic Non-coding RNAs Related to Mouse Liver Development. Mol. Genet. Genomics 289 (6), 1225–1235. 10.1007/s00438-014-0882-9 25012394

[B35] Martin G. G. Atshaves B. P. McIntosh A. L. Mackie J. T. Kier A. B. Schroeder F. (2008). Liver Fatty Acid-Binding Protein Gene-Ablated Female Mice Exhibit Increased Age-dependent Obesity. J. Nutr. 138 (10), 1859–1865. 10.1093/jn/138.10.1859 18806093 PMC2835297

[B36] Minoru K. Susumu G. Shuichi K. Yasushi O. Masahiro H. J. N. A. R. (2004). The KEGG Resource for Deciphering the Genome. Nucleic Acids Res. 32 (Database issue), D277–D280. 10.1093/nar/gkh063 14681412 PMC308797

[B37] Moon Y.-A. Hammer R. E. Horton J. D. (2009). Deletion of ELOVL5 Leads to Fatty Liver through Activation of SREBP-1c in Mice. J. Lipid Res. 50 (3), 412–423. 10.1194/jlr.M800383-JLR200 18838740 PMC2638104

[B38] Nicotra A. Pierucci F. Parvez H. Senatori O. (2004). Monoamine Oxidase Expression during Development and Aging. Neurotoxicology 25 (1-2), 155–165. 10.1016/S0161-813X(03)00095-0 14697890

[B39] Ohno Y. Suto S. Yamanaka M. Mizutani Y. Mitsutake S. Igarashi Y. (2010). ELOVL1 Production of C24 Acyl-CoAs Is Linked to C24 Sphingolipid Synthesis. Proc. Natl. Acad. Sci. U.S.A. 107 (43), 18439–18444. 10.1073/pnas.1005572107 20937905 PMC2973002

[B40] Pang X. Tang C. Guo R. Chen X. (2022). Non-cytochrome P450 Enzymes Involved in the Oxidative Metabolism of Xenobiotics: Focus on the Regulation of Gene Expression and Enzyme Activity. Pharmacol. Ther. 233, 108020. 10.1016/j.pharmthera.2021.108020 34637840

[B41] Parkinson A. Ogilvie B. W. Buckley D. B. Kazmi F. Parkinson O. J. C. Poisons D. S. T. T. B. S. O. (2001). Biotransformation of Xenobiotics.

[B42] Peng L. C. Piekos S. L. Guo G. Zhong X.-b. (2017). Role of Farnesoid X Receptor in the Determination of Liver Transcriptome during Postnatal Maturation in Mice. Nucl. Recept. Res. 4. 10.11131/2017/101308 PMC5962295 29795774

[B43] Peng L. Yoo B. Gunewardena S. S. Lu H. Klaassen C. D. Zhong X.-b. (2012). RNA Sequencing Reveals Dynamic Changes of mRNA Abundance of Cytochromes P450 and Their Alternative Transcripts during Mouse Liver Development. Drug Metab. Dispos. 40 (6), 1198–1209. 10.1124/dmd.112.045088 22434873 PMC3362789

[B44] Petrovic V. M. Janic-Sibalic V. Cvijic G. (1991). Development of Monoamine Oxidase Activity during Postnatal Development of the Ground Squirrel (Citellus Citellus). Comp. Biochem. Physiol. C Comp. Pharmacol. Toxicol. 98 (2-3), 377–380. 1676952

[B45] Prisingkorn W. Prathomya P. Jakovlić I. Liu H. Zhao Y. H. Wang W. M. (2017). Transcriptomics, Metabolomics and Histology Indicate that High-Carbohydrate Diet Negatively Affects the Liver Health of Blunt Snout Bream (*Megalobrama amblycephala*). Bmc Genomics 18, 856. 10.1186/s12864-017-4246-9 29121861 PMC5680769

[B46] Rolf A. Amos B. Wu C. H. Barker W. C. Brigitte B. Serenella F. (2004). UniProt: the Universal Protein Knowledgebase. Nucleic Acids Res. 32 (Database issue), D115–D119. 10.1093/nar/gkh131 14681372 PMC308865

[B47] Salini M. J. Turchini G. M. Wade N. M. Glencross B. D. (2015). Rapid Effects of Essential Fatty Acid Deficiency on Growth and Development Parameters and Transcription of Key Fatty Acid Metabolism Genes in Juvenile Barramundi (*Lates calcarifer*). Br. J. Nutr. 114 (11), 1784–1796. 10.1017/S0007114515003529 26411329

[B48] Saura J. Richards J. G. Mahy N. (1994). Differential Age-Related Changes of Mao-A and Mao-B in Mouse Brain and Pe Peripheral Organs. Neurobiol. Aging 15 (4), 399–408. 10.1016/0197-4580(94)90071-x 7969716

[B49] Storey J. D. Tibshirani R. (2003). Statistical Significance for Genomewide Studies. Proc. Natl. Acad. Sci. U.S.A. 100 (16), 9440–9445. 10.1073/pnas.1530509100 12883005 PMC170937

[B50] Sun J. Wu W. Y. Xu X. X. Ji H. (2021). DGAT1 Protects against Lipid Induced-Hepatic Lipotoxicity in Grass Carp (*Ctenopharyngodon idellus*). Aquaculture 534, 736328. 10.1016/j.aquaculture.2020.736328

[B51] Tatusov R. L. Galperin M. Y. Natale D. A. Koonin E. V. J. N. A. R. (2000). The COG Database: a Tool for Genome-Scale Analysis of Protein Functions and Evolution. Nucleic Acids Res. 28 (1), 33–36. 10.1093/nar/28.1.33 10592175 PMC102395

[B52] Tayama Y. Moriyasu A. Sugihara K. Ohta S. Kitamura S. (2007). Developmental Changes of Aldehyde Oxidase in Postnatal Rat Liver. Drug Metabolism Pharmacokinet. 22 (2), 119–124. 10.2133/dmpk.22.119 17495419

[B53] Tipton K. F. Boyce S. O'Sullivan J. Davey G. P. Healy J. (2004). Monoamine Oxidases: Certainties and Uncertainties. Curr. Med. Chem. 11 (15), 1965–1982. 10.2174/0929867043364810 15279561

[B54] Torrecillas S. Caballero M. J. Mompel D. Montero D. Zamorano M. J. Robaina L. (2017). Disease Resistance and Response against Vibrio Anguillarum Intestinal Infection in European Seabass ( *Dicentrarchus labrax* ) Fed Low Fish Meal and Fish Oil Diets. Fish Shellfish Immunol. 67, 302–311. 10.1016/j.fsi.2017.06.022 28602741

[B55] Veerkamp J. H. van Kuppevelt T. H. M. S. M. Maatman R. G. H. J. Prinsen C. F. M. (1993). Structural and Functional Aspects of Cytosolic Fatty Acid-Binding Proteins. Prostagl. Leukot. Essent. Fat. Acids 49 (6), 887–906. 10.1016/0952-3278(93)90174-u 8140117

[B56] Vyskočilová E. Szotáková B. Skálová L. Bártíková H. Hlaváčová J. Boušová I. (2013). Age-related Changes in Hepatic Activity and Expression of Detoxification Enzymes in Male Rats. BioMed Res. Int. 2013, 408573. 10.1155/2013/408573 23971034 PMC3736498

[B57] Wang L. Zhu P. Mo Q. Luo W. Du Z. Jiang J. (2022). Comprehensive Analysis of Full-Length Transcriptomes of Schizothorax Prenanti by Single-Molecule Long-Read Sequencing. Genomics 114, 456–464. 10.1016/j.ygeno.2021.01.009 33516848

[B58] Wang X. Yang L. Wang Y.-C. Xu Z.-R. Feng Y. Zhang J. (2020). Comparative Analysis of Cell Lineage Differentiation during Hepatogenesis in Humans and Mice at the Single-Cell Transcriptome Level. Cell Res. 30 (12), 1109–1126. 10.1038/s41422-020-0378-6 32690901 PMC7784864

[B59] Waxman D. J. (1999). P450 Gene Induction by Structurally Diverse Xenochemicals: Central Role of Nuclear Receptors CAR, PXR, and PPAR. Archives Biochem. Biophysics 369 (1), 11–23. 10.1006/abbi.1999.1351 10462436

[B60] Wu G. Bazer F. W. Burghardt R. C. Johnson G. A. Kim S. W. Knabe D. A. (2011). Proline and Hydroxyproline Metabolism: Implications for Animal and Human Nutrition. Amino Acids 40 (4), 1053–1063. 10.1007/s00726-010-0715-z 20697752 PMC3773366

[B61] Wu K. C. Cui J. Y. Liu J. Lu H. Zhong X.-b. Klaassen C. D. (2019). RNA-seq Provides New Insights on the Relative mRNA Abundance of Antioxidant Components during Mouse Liver Development. Free Radic. Biol. Med. 134, 335–342. 10.1016/j.freeradbiomed.2019.01.017 30659941 PMC6588412

[B62] Wu S. Liu Y. Guo W. Cheng X. Ren X. Chen S. (2018). Identification and Characterization of Long Noncoding RNAs and mRNAs Expression Profiles Related to Postnatal Liver Maturation of Breeder Roosters Using Ribo-Zero RNA Sequencing. BMC Genomics 19 (1), 498. 10.1186/s12864-018-4891-7 29945552 PMC6020324

[B63] Xie C. Mao X. Huang J. Ding Y. Wu J. Dong S. (2011). KOBAS 2.0: a Web Server for Annotation and Identification of Enriched Pathways and Diseases. Nucleic Acids Res. 39 (Web Server issue), W316–W322. 10.1093/nar/gkr483 21715386 PMC3125809

[B64] Xu E. Zhang L. Yang H. Shen L. Feng Y. Ren M. (2019). Transcriptome Profiling of the Liver Among the Prenatal and Postnatal Stages in Chickens. Poult. Sci. 98 (12), 7030–7040. 10.3382/ps/pez434 31376353 PMC8913967

[B65] Yong B. Dawson T. L. Jr. Chen Y. T. J. G. (1996). Human Glycogen Debranching Enzyme Gene (AGL): Complete Structural Organization and Characterization of the 5' Flanking Region. 38(2)**,** 155–165. 10.1006/geno.1996.0611 8954797

[B66] Yun K. U. Oh S. J. Oh J. M. Kang K. W. Myung C.-S. Song G. Y. (2010). Age-related Changes in Hepatic Expression and Activity of Cytochrome P450 in Male Rats. Arch. Toxicol. 84 (12), 939–946. 10.1007/s00204-010-0520-1 20130842

